# Quantitative Assessment of Traumatic Upper-Limb Peripheral Nerve Injuries Using Surface Electromyography

**DOI:** 10.3389/fbioe.2020.00795

**Published:** 2020-07-17

**Authors:** Weidi Tang, Xu Zhang, Yong Sun, Bo Yao, Xiang Chen, Xun Chen, Xiaoping Gao

**Affiliations:** ^1^School of Information Science and Technology, University of Science and Technology of China, Hefei, China; ^2^Institute of Criminal Sciences, Hefei Public Security Bureau, Hefei, China; ^3^Institute of Biomedical Engineering, Chinese Academy of Medical Sciences and Peking Union Medical College, Tianjin, China; ^4^Department of Rehabilitation Medicine, The First Affiliated Hospital of Anhui Medical University, Hefei, China

**Keywords:** clinical assessment, peripheral nerve injury, surface electromyography, non-invasive examination, machine learning

## Abstract

**Background:**

There is a great demand for convenient and quantitative assessment of upper-limb traumatic peripheral nerve injuries (PNIs) beyond their clinical routine. This would contribute to improved PNI management and rehabilitation.

**Objective:**

The aim of this study was to develop a novel surface EMG examination method for quantitatively evaluating traumatic upper-limb PNIs.

**Methods:**

Experiments were conducted to collect surface EMG data from forearm muscles on both sides of seven male subjects during their performance of eight designated hand and wrist motion tasks. All participants were clinically diagnosed as unilateral traumatic upper-limb PNIs on the ulnar nerve, median nerve, or radial nerve. Ten healthy control participants were also enrolled in the study. A novel framework consisting of two modules was also proposed for data analysis. One module was first used to identify whether a PNI occurs on a tested forearm using a machine learning algorithm by extracting and classifying features from surface EMG data. The second module was then used to quantitatively evaluate the degree of injury on three individual nerves on the examined arm.

**Results:**

The evaluation scores yielded by the proposed method were highly consistent with the clinical assessment decisions for three nerves of all 34 examined arms (7 × 2 + 10 × 2), with a sensitivity of 81.82%, specificity of 98.90%, and significate linear correlation (*p* < 0.05) in quantitative decision points between the proposed method and the routine clinical approach.

**Conclusion:**

This study offers a useful tool for PNI assessment and helps to promote extensive clinical applications of surface EMG.

## Introduction

The term peripheral nerve injuries (PNIs) refers to a clinical condition caused by ischemia-reperfusion or damage of the peripheral nerves, that include torso and limb sensory, motor, and nerve autonomic dysfunction. Among PNIs, traumatic upper-limb PNI is relatively common (occurring on the upper-limb after a penetrating injury, crush, stretch, ischemia, or other traumatic injuries) (Noble et al., 1998; Campbell, 2008). People with traumatic upper-limb PNI usually suffer from sensory disturbance, dyskinesia, muscle atrophy in the control area of the damaged nerves, and have a reduced quality of life (Selecki et al., 1982). For example, those with radial nerve injury typically suffer from a weakness of the wrist and finger extensor muscles and show carpoptosis (Sallomi et al., 1998). The evaluation of upper-limb PNI has great importance for clinical treatment and rehabilitation guidance, as well as social and judicative significance. For insurance claims, as an example, the amount of compensation depends on the degree of disability following the PNI. Especially in judicial expertise, the resultant degree of the PNI is an important basis for sentencing who causes the PNI.

Currently, the clinical evaluation of peripheral nerve injury relies primarily on clinical history, clinical symptoms, and physical/neurological examination (Aminoff, 2012; Zeidenberg et al., 2015). Clinical symptoms usually include neuromuscular physiology state, special body posture, motor and sensory function, and reflex issues (Sallomi et al., 1998). More objective information can be obtained by physical/neurological examination. In previous studies, many diagnostic methods have been successfully used for PNI evaluation, such as invasive/needle electromyography (EMG) examination, MRI, and high-resolution ultrasonography (HRU) (Sunderland, 1968; Chang et al., 2019). Direct imaging with MRI or HRU is successful in clearly and accurately demonstrating neural integrity. Moreover, invasive/needle EMG can detect nerve integrity by neuromuscular activities. Through electrical stimulation, the evoked motor unit action potentials (MUAPs) are recorded to reflect potential abnormality according to nerve conduction velocity, EMG amplitude, and other signal morphological features (Robinson, 2000). However, all these methods rely on invasive/painful protocols or specialized equipment to be manipulated by clinical professionals, which is a rigorous condition that hinders their pervasive applications. The strong subjectivity of the examiners involved in the interpretation of the examination results is another problem. Therefore, a convenient and practical protocol along with an automatic expert system involving intelligent data processing and machine learning algorithms is required for quantitative and objective PNI evaluation.

Compared with invasive EMG examination, surface EMG (sEMG) is an alternative approach that detects neuromuscular activities from the skin surface in a non-invasive manner. The electrode placed over the skin surface is not as selective as that of the invasive needle. Therefore, the sEMG signal appears in interference patterns due to the superposition of a large number of MUAP waveforms. This is the primary reason for restricting clinical applications of the sEMG. To take advantage of its non-invasive feature, many studies have focused on the sEMG examination of various neuromuscular diseases, including spinal cord injury (Sherwood et al., 1996; Alexander et al., 2009), stroke (Kallenberg and Hermens, 2009; Li et al., 2017), cerebral palsy (Steele et al., 2015; Tang et al., 2015; Cappellini et al., 2016), ALS (Zhang et al., 2013a), dysphagia, and odynophagia (Vaiman and Eviatar, 2009). Its advantages in both non-invasive and ease of operation make it suitable for long-term and repetitive PNI monitoring, especially toward home or community rehabilitation. In addition, besides the nerve integrity, the sEMG was reported to have the capability to evaluate motor functions, which is also of great importance for PNI assessment and management. Moreover, the fast operation property of the surface EMG approach can facilitate early judicial intervention to PNI cases for grassroots police, without waiting for transfer to the clinician or forensic experts.

Based on the above considerations, in this study, we proposed a novel framework for evaluating upper-limb traumatic PNIs using surface EMG. To our knowledge, this is the first attempt to apply the sEMG to the assessment of traumatic upper-limb PNI. The proposed method includes intelligent signal processing and machine learning procedures, which provide a new automatic and objective solution for the PNI assessment. We expect that these advances will help to expand the usability of PNI evaluation from routine medical diagnosis to many special occasions, such as home or community rehabilitation guidance and judicial intervention.

## Materials and Methods

### Subjects

Seven subjects with upper-limb PNIs (labeled as S1–S7, all males, age: 24 ± 6.6 years, mean ± standard deviation, range 17–34 years) and 10 age- and gender-matched healthy control subjects (C1–C10, age: 22 ± 4 years, range 17–29 years, male) were recruited for data collection experiments in this study. The study was approved by the Medical Ethics Review Committee of The First Affiliated Hospital of Anhui Medical University. Inclusion criteria for patients include: (1) experience of a unilateral PNI caused by trauma, at either the radial nerve, median nerve, or ulnar nerve. Subjects with simultaneous injuries of multiple nerves were also included when; (2) at least 6 months had passed since the onset of the injury; (3) the patient was in a stable condition with all wounds healing; and (4) there was no history of any neuromuscular disease, except the injury. Written consent was obtained from all subjects before the experiments.

All patients were tested through a clinical, electrophysiological examination on their radial nerves, median nerves, and the ulnar nerves using needle EMG. This approach routinely reported a three-graded degree of injury for each nerve on both sides: “−” indicates no injury; “+” indicates mild injury; and “++” indicates severe injury (likely involving nerve rapture). Detailed information of all the patients and their clinical examination reports are shown in Table 1.

**TABLE 1:** Physical information and clinical assessment decisions for all subjects with peripheral nerve injuries.

**ID #**	**Age range (year)**	**Gender**	**Side of the injury**	**Clinical assessment decisions**					
				**Left**			**Right**		
				**Ulnar**	**Median**	**Radial**	**Ulnar**	**Median**	**Radial**
S1	26–30	M	L	+ ⁣ +	−	−	−	−	−
S2	31–35	M	R	−	−	−	+	+	+
S3	16–20	M	L	−	−	+ ⁣ +	−	−	−
S4	16–20	M	L	+ ⁣ +	+ ⁣ +	−	−	−	−
S5	31–35	M	L	−	−	+ ⁣ +	−	−	−
S6	16–20	M	R	−	−	−	+ ⁣ +	+ ⁣ +	−
S7	21–25	M	L	−	+	−	−	−	−

*“−” indicates health; “+” indicates an injury; “++” indicates a severe injury*

### Experiments

**FIGURE 1:**
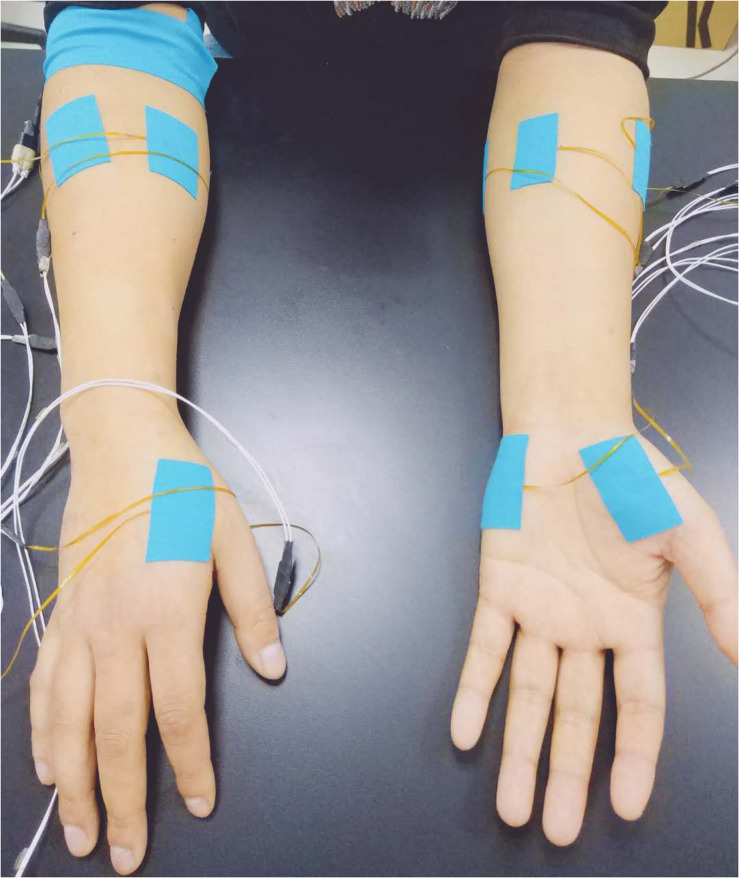
Electrode placement for surface EMG data recording.

Fourteen surface EMG sensors were used to collect data from three hand muscles and four forearm muscles on both sides of all subjects. Each EMG sensor has two round electrode probes, with a 3-mm diameter for each probe and an 8-mm center distance between them, constituting a single-differential surface EMG data recording channel. The hand muscles include the abductor pollicis brevis (APB) muscle, the first dorsal interosseous (FDI) muscle, and the abductor digitiminimi (ADM) muscle. On each side of the forearm, four sensors were placed around the circumference of the forearm, at a position of 25% of the entire distance from the elbow to the wrist. These were equally spaced and placed over the ulnar side, the anterior side, the radial side, and the posterior side of the forearm, mainly targeting the ulnar flexor carpi, long palmar, extensor carpi radialis, and extensor of fingers, respectively (Figure 1). Considering the complicated structural distribution of the forearm muscles and their possible cross-talks, activities from a variety of nearby muscles could also be sensed. Since these muscles were innervated by the three nerves tested in this study, their surface EMG activities were intentionally included for the following analysis. After the skin preparation with medical alcohol, each surface EMG sensor was firmly attached to each targeted muscle/position, with its electrode pair along the direction of muscle fibers. As a reference, a round electrode was placed on the right arm fossa cubitalison.

**FIGURE 2:**
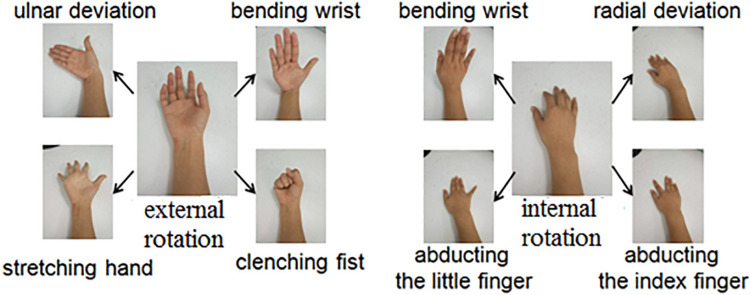
Illustration of eight designated motion tasks.

During the experiment, subjects were seated in a comfortable chair with their arms relaxed on a height-adjusted table. Each subject was instructed to perform eight tasks from the initial relaxation/rest state: ulnar deviation, clenching fist, stretching hand, bending of the wrist with external rotation of elbow joint and perform radial deviation, abducting the index finger, abducting the little finger, and bending of the wrist with internal rotation of elbow joint (Figure 2). Subjects were asked to perform these tasks with both arms/sides simultaneously. Subjects were instructed to perform each task with a maximal voluntary contraction (MVC) and to hold it as stably as possible for at least 3 s. In this study, the MVC represented a condition when the subject performed muscle contractions with maximal efforts. Although the actual muscle contraction strength/force was not precisely measured during the experiments, the surface EMG recordings were real-time monitored, and the subjects were encouraged to produce as high level of EMG intensity/amplitude as possible during their task performances, so as to ensure the quality of MVC. To gain a sufficient amount of data, each task was repeated at least three times in one trial. To avoid mental and muscular fatigue, sufficient rest was allowed between two consecutive trials.

Surface EMG data were recorded for every task with a custom-made data acquisition system supporting up to 128 EMG channels. This system has been validated and successfully applied in various neuroscience and engineering studies involving electrophysiological data recording (Tang et al., 2015, 2018). Each recorded EMG channel was amplified by a two-stage amplifier with a total gain of 52dB, band-pass filtered at 16–610 Hz, and subsequently converted into digitalized data with an 18-bit A/D converter. The sampling rate for each channel was set to 1 kHz. All recorded data were transferred into a laptop computer via a USB cable for off-line analysis in the Matlab (The Mathworks, MA, United States) environment with customized programs.

### Data Preprocessing

Each channel of surface EMG was pre-processed by a zero-lag fourth-order Butterworth band-pass filter at 20–500 Hz to eliminate low-frequency motion artifacts and high-frequency interferences. If necessary, a set of second-order notch filters at the 50-Hz power line interference and its harmonics were also applied.

The recorded EMG data showed three muscular activity bursts corresponding to three repetitions of each task in one trial. This phenomenon was mainly observed from the seven EMG channels for the tested arm performing the tasks. A data segment of 3-s EMG activity was selected for each muscular activity burst, and thus three data segments were produced for each task. The EMG signals in the form of seven channels in each data segment were further divided into a series of non-overlapping analysis windows with a window length of 100 ms. We chose such a windowing approach to produce a sufficient number of data samples, while these windows were not overlapped to maintain the diversity of resultant data samples. Finally, 90 analysis windows/samples were obtained for each tested arm performing each task. These analysis windows were also considered as basic data samples in the following feature extraction and pattern classification analyses.

A great many studies have been performed for developing features to characterize the raw surface EMG data for a pattern classification purpose (Phinyomark et al., 2012). Among these, Hudgins’ time-domain (TD) features (Englehart and Hudgins, 2003), autoregressive (AR) model coefficients (Zeng et al., 2019), and time-dependent power spectrum descriptors (TD-PSDs) (Altimemy et al., 2016) have been popularly used and achieved successful applications. Some studies also believe that there is little difference in the effectiveness of these features in practical use (Li et al., 2016). According to some pre-tests for these features and their combination, the set of four TD features was used in our study, including the mean absolute value (MAV), waveform length (WL), number of zero-crossings (ZC), and number of slope sign changes (SSC). Its reported effectiveness in surface EMG pattern recognition was another reason for adopting the TD feature set (Zhang et al., 2017). The four TD features were calculated separately for each sample or channel.

**FIGURE 3:**
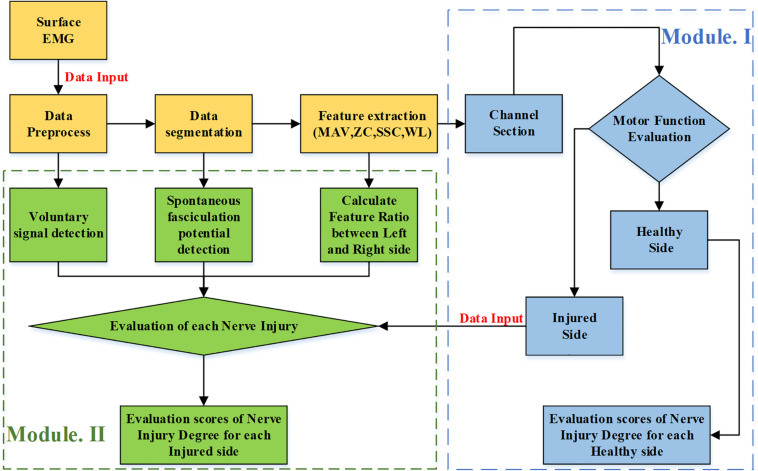
The framework for evaluating injury degrees of three individual nerves on the forearm in the proposed method.

Subsequent data analysis consisted of two modules: Module I makes the judgment about whether the tested arm is affected by PNI. On this basis, if any nerve injury was judged, Module II evaluated injury degrees of three individual nerves (the radial nerve, median nerve, and ulnar nerve) by quantitative scores. Figure 3 illustrates the framework for traumatic upper-limb PNI assessment using surface EMG data processing.

### Module I: Arm Injury Judgment

This module was designed to make a brief judgment on whether any nerve is injured in the tested arm. Therefore, it was applied to solve a general two-class problem: normality or injury. Given the data recorded from the tested arm executing all tasks, the tested arm could be judged as being injured as long as an exception (with respect to the normality) was presented anytime and anywhere (at any channel during the performance of any task).

For each tested arm of a subject, a total of 8 (tasks) × 90 (samples per task) × 7 (channels per sample) × 4 (features per channel) features were extracted, from which only a few features needed to be selected (because the majority were believed to carry irrelevant or redundant information). It was assumed that discriminative information associated with the nerve injury could be examined from a specific location, which represents a combination of an examined muscle and a task corresponding to the muscle function. Thus, a feature selection procedure was conducted to choose a subset of features from the entire set of 224 (8 motions × 7 channels × 4 features) different kinds of features. Here, the 90 samples were viewed as 90 repetitive measures of each kind of feature. To determine the best feature subset, Fisher’s class separability index (FCSI) (Englehart, 1998) was employed as the discriminant measure, which is described as:

(1) $$\text{FCSI}=\sum_{\text{a}=1}^{\text{C}-1}\sum_{\text{b}=\text{a}+1}^{\text{C}}\frac{{\left|{\begin{array}{c} \_\,\_\\ m \end{array}}_{\text{j},\text{c}}^{\left(\text{a}\right)}-{\begin{array}{c} \_\,\_\\ m \end{array}}_{\text{j},\text{c}}^{\left(\text{b}\right)}\right|}^{2}}{\begin{array}{c} \text{var}\\ 1\leq \text{i}\leq {\text{N}}_{\text{a}} \end{array}\,\left({\text{x}}_{\text{i},\left(\text{j},\text{k}\right)}^{\left(\text{a}\right)}\right)\,+\begin{array}{c} \text{var}\\ 1\leq \text{i}\leq {\text{N}}_{\text{b}} \end{array}\,\left({\text{x}}_{\text{i},\left(\text{j},\text{k}\right)}^{\left(\text{b}\right)}\right)}$$

where *a* and *b* represent the indices of two different classes (injury or normality), and *c* represents the number of different classes. Generally, a higher value of FCSI indicates a higher degree of class separability. This feature selection algorithm is able to rank the features in descending order of their FCSI values and make it practical to choose a subset of features with top N FCSI values as being the most discriminative features (Wang et al., 2016).

After the feature selection, an N-dimensional feature vector was formed for each sample, and there were 90 samples for each tested arm. A conventional linear discriminant classifier (LDC) was used for classification between normality and injury (Smola et al., 2000; Webb, 2003; Zhang H. et al., 2013) because of its satisfactory performance and high practicability for surface EMG classification (Englehart et al., 1999). The implementation of the LDC is to construct a linear classifier by modeling the within-class density of each class as a multi-variant Gaussian distribution (Mika et al., 1999). For each tested arm, the classifier produced 90 decisions corresponding to its 90 samples. If 95% of these samples had a decision of nerve injury, we, therefore, identified a nerve injury for the tested arm; and otherwise, the tested arm was identified to be normal. Specifically, both arms of any subject with SCI were tested independently.

A leave-one-out cross-validation scheme was used to train the classifier and then to make a judgment of nerve injury for the tested arm. The data from all arms except one arm were used for determining the selected feature subset and training the classifier. Therefore, the data from the remaining arm were used for testing. This procedure was repeated 34 times (34 arms in total were tested in the experiments) so as to ensure each arm’s data were considered as the testing dataset once. The performance of nerve injury judgment was evaluated by accuracy, which was defined as the percentage of correctly identified arms to all the arms tested in this study. Specifically, we intentionally adjusted the number *N* of selected subset features from 1 to 10, and *N* was finally determined to be two due to its optimal and satisfactory performance.

### Module II: Evaluation of Injury Degree for Each Nerve

Given the decision of nerve injury from Module I, this module was designed to further evaluate the degree of the injury for individual nerves in the tested arms. Three nerves were considered in each tested arm: the ulnar, medial, and radial nerves. To determine their degrees of injury, we had to consider anatomical knowledge, including their dominated muscles and muscle functions (Selecki et al., 1982; Noble et al., 1998). In detail, the ulnar nerve function can be represented by the EMG channel from ADM muscle during the performance of the task ulnar deviation (UD); the median nerve function is related to the EMG channel from APB muscle during the performance of the task radial deviation (RD); and the radial nerve function is associated with the EMG channel from the APB muscle and the radial side muscle (RM) during the performance of the task RD. Therefore, the examination of any individual nerve relied on the data from the corresponding channel/muscle and the corresponding task. The degrees of injury of these nerves were evaluated from three different aspects, including neural control command delivery to produce voluntary EMG signals, the presence of abnormal involuntary EMG activity, and comparison between arms on both sides of one subject.

We applied an accumulation deduction system to quantify the degree of injury evaluation for each nerve. Initially, each nerve has 10 points, indicating no injury and its intactness. Any phenomenon associated with a nerve injury revealed by the following three-aspect evaluation approach is marked by a deduction of points. The number of deducted points varies across reported phenomena, indicating different injury degrees. The point of one nerve can be deducted into 0, representing the severest injury and the least function.

#### Step 1: Examination of Sufficient Voluntary EMG Activities

The generation of sufficient EMG activities during voluntary muscle contractions is a good indication of an intact motor command delivery pathway. Such EMG generation capability might be affected by the PNI due to the hampered delivery of motor commands, further contributing to impaired motor function (Noble et al., 1998; Campbell, 2008). The examination of voluntary EMG mainly relied on the detection of its onset and offset during the performance of the designated task. Therefore, in our study, a routine approach for EMG onset/offset detection was employed and modified accordingly.

The examination of voluntary EMG activities was only performed on specific combinations of the EMG channel/muscle and task, which were designated above. Given a specific channel of EMG recorded during task performance in one trial, this examination included five steps: (1) Calculate the signal energy defined as the square of signal amplitude and filter it with a sliding window averaging using a window length of ten samples (the current sample plus nine previous samples); (2) Define an energy threshold as 5-times the quiescent baseline energy; (3) Detect the onset as the time of signal energy climbing up across the threshold; similarly, detect the offset as the time of signal energy falling below the threshold; (4) Calculate the voluntary EMG time duration by the offset time minus the onset time; and (5) Produce a decision for this examination: normality or abnormality when the duration time was more than 3-s or not, respectively.

The examination outcomes of sufficient voluntary EMG activities have to be expressed as quantitative deduction points according to the accumulation deduction system. Table 2 clearly describes items for nerve injury judgment under various conditions. These items for revealing whether an individual nerve was injured or not were derived from anatomic knowledge regarding the muscle nerve innervation relationship and corresponding neuromuscular functions. According to Table 2, the nerve injury decision can be made, and the nerve with a decision of injury gets −5 points in this aspect of the examination.

**TABLE 2:** The relationship between PNI decisions on individual nerves and different conditions.

**Conditions**		**Injury Decisions**	
**APB muscle in RD task**	**RM muscle in RD task**	**Radial nerve**	**Medial nerve**
o	o	none	none
o	x	injury	none
x	o	none	injury
x	x	injury	none
	
**ADM muscle in UD task**		**Ulnar nerve**	
o		none	
x		injury	

#### Step 2: Detection of Involuntary EMG Activities

The appearance of involuntary EMG has often been reported and is considered a typical pattern of abnormal EMG activities after nerve injury. These involuntary EMG activities can be grossly divided into two cases that can be detected via surface EMG. One case is spontaneous EMG activities, mainly appearing as spontaneous fasciculation potentials sporadically distributed within the quiescent baseline when the muscle is supposed to be relaxed. The fasciculation potential is regarded as an abnormal EMG pattern due to denervation of motor units or a group of muscle fibers in the examined muscle (de Carvalho and Swash, 1998). The detection of spontaneous fasciculation has been a gold-standard criterion for the diagnosis of amyotrophic lateral sclerosis (Chen and Zhou, 2014), while its occurrence has also been reported in PNI (Hermens et al., 1984). The other is spastic muscular activities, appearing as the fact that the muscle failed to be voluntarily relaxed, but its motor units continue to discharge repetitively to produce a series of relatively larger action potentials. Although spastic activities can only be attributed to the hyper-activity of upper motor neurons (muscle spasticity seldom occurs after PNI), its electrophysiological appearance in EMG data is very similar to that of spontaneous fasciculation potentials. Therefore, we used the same approach to detect either of them. Such involuntary EMG activity was evidently the case (i.e., spontaneous fasciculation potentials) in this study.

The approach for the detection of involuntary activity consisted of four steps: (1) Calculate the signal energy, which is regarded as the square of amplitude; (2) Filter the signal energy curve using a smoothing window length of 10 data points, where any sporadical spike (i.e., an involuntary action potential) was identified as a peak; (3) Detect these peaks by selecting data points with the top 3% filtered energy values. If more than one peak was located within a window of 10 ms, only the largest was regarded as the peak; (4) Confirm these sporadic peaks by comparing each peak with nearby data points in a time deviation of 40 ms beside the peak. If the peak was 20-times larger than any of the nearby data, this peak could be confirmed. Any signal segment with more than one detected sporadic peak was regarded to be abnormal in terms of carrying involuntary EMG activities.

The examination outcomes of involuntary EMG activities have to be expressed as quantitative deduction points according to the accumulation deduction system. We used the same Table 2 to transfer examination outcomes to decisions of injury on three examined nerves. According to Table 2, any nerve with a decision of injury gets −5 points in this aspect of the examination.

#### Step 3: Calculation of Muscle Activation Ratio Between Both Sides

Given the side dependency of PNI, an intra-subject bilateral comparison was an important indicator that helps to overcome potential cross-subject variability. It was especially useful in certain cases to reveal PNI abnormality when the above two aspects of examination failed to report any abnormality. The bilateral comparison was performed to reveal potential abnormality at the individual nerve level. We calculated the average of four features mentioned above for three segments in each trial (the four statistics were calculated from the signal in an entire segment instead of 90 analysis windows). To eliminate the influence of individual differences, we calculated the ratio of both sides (subjects were asked to perform these tasks with both arms/sides simultaneously in the experiments). For example, this ratio for the WL of APB muscle in the motion task RD was calculated as:

(2) $$\text{R}\,\_\,\text{WL}\,\_\,\text{APB}\,\_\,\text{RD}=\frac{\text{WL}\,\_\,\text{APB}\,\_\,\mathrm{RD}\,\mathrm{on}\,\mathrm{the}\,\mathrm{left}\,\mathrm{side}}{\text{WL}\,\_\,\text{APB}\,\_\,\mathrm{RD}\,\mathrm{on}\,\mathrm{the}\,\mathrm{right}\,\mathrm{side}}$$

These ratios were supposed to be within 0 and 1. To ensure the ratio between 0 and 1, if any ratio was greater than 1, its reciprocal was used instead. Similarly, to evaluate a corresponding nerve injury, a ratio was just calculated for any designated combination of muscle and task. Table 2 was also used to determined which nerve is injured based on the abnormality determined by the ratio. For an individual nerve, the calculated ratio had to be expressed as quantitative deduction points according to the accumulation deduction system. As a result, some minus points (from 1 to 5 minus points) were applied to an injured nerve. Table 3 lists the different degrees of the nerve injury quantified by the minus points according to the ratio in this aspect of the examination. Please note if both the APB muscle and the RM muscle reported abnormality during the motion task RD performance (Table 2), the more severely affected muscle was adopted for evaluating the radial nerve injury (Table 3).

**TABLE 3:** Deduction points according to the ratio.

Ratio(*r*)	0.25 < *r* ≤ 1	0.20 < *r* ≤ 0.25	0.15 < *r* ≤ 0.20	0.10 < *r* ≤ 0.15	0.05 < *r* ≤ 0.10	0 < *r* ≤ 0.05
Deduction points	0	−1	−2	−3	−4	−5

The proposed accumulation deduction system, consisting of the above three aspects, was used to quantify the degree of injury of each nerve. Initially, each nerve has 10 points, and no points were further deducted after reaching a 0 score. According to the three-grade clinical assessment, we also predefined three grades with our 10-point scale: the 10 points indicated *no* injury and its intactness, 7–9 points indicated a *mild* injury, and 0–6 points indicated a *severe* injury.

### Performance Evaluation

The validity of the proposed method for evaluating traumatic upper limb PNI was demonstrated by comparing the evaluation decisions of the routine clinical assessment and the proposed evaluation framework. We calculated the sensitivity, specificity, positive predictive value, negative predictive value, and Youden’s index for the proposed method, given the clinical assessment decisions as to the ground truth. The above indexes were described as:

(3) $$\text{sensitivity}=\,\frac{\text{TP}}{\text{TP}+\text{FN}}\times 100\%$$

(4) $$\text{specificity}=\,\frac{\text{TN}}{\text{FP}+\text{TN}}\times 100\%$$

(5) $$\mathrm{positive}\,\mathrm{predictive}\,\mathrm{value}=\,\frac{\text{TP}}{\text{TP}+\text{FP}}\times 100\%$$

(6) $$\mathrm{negative}\,\mathrm{predictive}\,\mathrm{value}=\frac{\text{TN}}{\text{FN}+\text{TN}}\times 100\%$$

(7) $${\mathrm{Youden}}^{\prime }\,s\,\mathrm{Index}=\text{sensitivity}+\text{specificity}-1$$

where TP represents the number of injured arms/nerves correctly diagnosed as injured, FP represents the number of healthy arms/nerves wrongly diagnosed as injured, FN represents the number of injured arms/nerves wrongly diagnosed as healthy, and TN represents the number of healthy arms/nerves correctly diagnosed as healthy.

We also performed a series of regression analyses to compare the injury degree of each nerve between the clinical assessment approach and the proposed method. The three-grade clinical assessment decisions (“−”, “++”, “++”) were expressed quantitatively as 0, −1, and −2. The regression analyses between clinical assessment decisions and our evaluation points were conducted for each nerve. The level of statistical significance was set to *p* < 0.05 for all analyses. All statistical analyses were completed using SPSS software (ver. 16.0, SPSS Inc., Chicago, IL, United States).

## Results

### Classification Results Between Arm Nerve Normality and Injury

**FIGURE 4:**
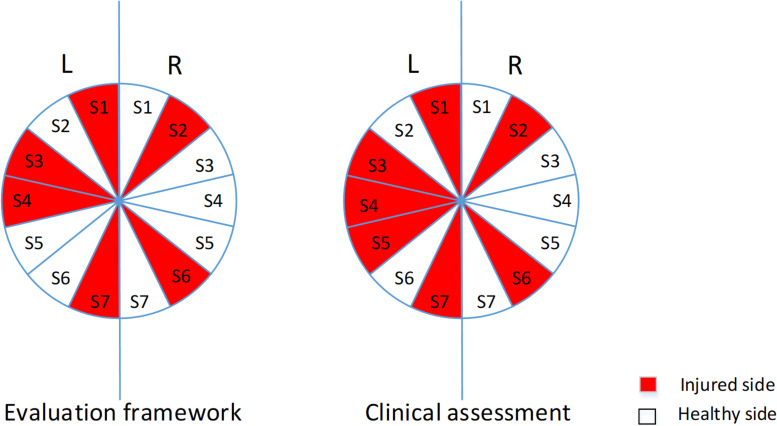
The pie graph of evaluation decisions and clinical assessment decisions for both arms of subjects with PNIs. The left and right arms of S1–S7 were represented by a 25.71–degree fan and a fan in dark indicated that any PNI is identified on that arm.

Figure 4 shows the results for general arm injury identification for all subjects with PNIs. The proposed method yielded almost the same decisions as to the clinical assessment, except for the left arm of subject five. For healthy subjects, both sides of all ten healthy subjects were correctly determined to be normal and healthy. Therefore, when evaluating the performance of Module I in the proposed method, the sensitivity, specificity, positive predictive value, negative predictive value, and Youden’s index are 85.71, 100, 100, 96.43, and 85.71%, respectively.

### Evaluating Injury Degrees of Individual Nerves

#### Evaluation Results of Each Step

The results of injured arms in each step, including the detection of voluntary EMG signals and involuntary EMG activities for selected muscles and motions, and the range of selected features’ ratio, are shown in Table 4.

**TABLE 4:** Results of evaluating each nerve in each step of the Module II.

**Evaluation step**	**Muscle and task combination**	**S1**		**S2**		**S3**		**S4**		**S5**		**S6**		**S7**	
		**L**	**R**	**L**	**R**	**L**	**R**	**L**	**R**	**L**	**R**	**L**	**R**	**L**	**R**
Step1: voluntary signal detection	ADM in UD	0	/	/	0	0	/	0	/	0	0	/	−5	0	/
	APB in RD	0	/	/	0	0	/	0	/	0	0	/	0	0	/
	RM in RD	0	/	/	0	0	/	0	/	0	0	/	0	0	/
Step2: spontaneous fasciculation detection	ADM in UD	−5	/	/	0	0	/	−5	/	0	0	/	0	0	/
	APB in RD	0	/	/	0	0	/	−5	/	0	0	/	0	0	/
	RM in RD	0	/	/	0	0	/	0	/	0	0	/	0	0	/
Step3: range of ratio	ADM in UD	−3		−3		−2		−3		0		0		0	
	APB in RD	0		−3		−4		−5		0		−5		−2	
	RM in RD	0		0		−1		0		0		0		0	

*“/” indicates “not applicable”.*

In terms of examining sufficient voluntary EMG activities, a decision of normality was given for all examined muscles and motions. Only the right ADM muscle of subject 6 in task UD was found to be abnormal, so that 5 minus points were applied to the ulnar nerve on the right arm of the subject.

When detecting involuntary EMG activities, an abnormality was reported in three cases: the left ADM muscles in motion task UD for both subject 1 and subject 4, and the left APB muscle of the subject 4. No involuntary EMG activity was found for the remaining muscles from all subjects. Therefore, corresponding deductions points were applied to the ulnar nerve of subject 1 and the ulnar and medial nerves of subject 4.

When examining the difference between both arms, the abnormality was found in most cases, and corresponding deduction points were applied (Table 4).

#### Evaluation Points and Clinical Quantitative Results

Table 5 reports the final evaluation scores derived from the proposed method and the clinical assessment decisions for individual nerves on both sides of all subjects with PNIs. Evidently, all healthy subjects were diagnosed as being “healthy” on each nerve with full points using the proposed method. From Table 5, we can conclude that the proposed method was mostly consistent with the clinical assessment decisions.

**TABLE 5:** Evaluation scores from both clinical assessment and the proposed method for all three examined nerves of all subjects.

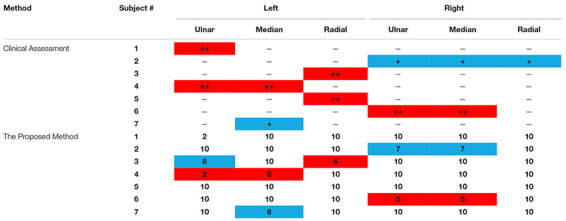

*The points marked with red indicate severe injury; The points marked with blue indicate mild injury; The points without any color indicate no injury or normality.*

Given the clinical assessment decision (injury or no injury) on each nerve as the ground truth, it is also practical to calculate the five metrics for evaluating the performance of the proposed methods. When each of three individual nerves from both sides of all subjects (including all healthy subjects and subjects with PNIs) were considered independently, the sensitivity, specificity, positive predictive value, negative predictive value, and Youden’s index were 81.82, 98.90, 90, 97.83, and 80.72%, respectively. Furthermore, the three-grade clinical assessment decision was quantified by scores 0, −1, and −2. Thus, in a regression analysis, the clinical assessment-derived evaluation scores and the proposed method were correlated (*p* < 0.05) when data from three nerves were pooled together. This was also true when data from each individual nerve was used (*p* < 0.05).

## Discussion

This paper presents a framework for quantitative evaluation of upper limb PNI using surface EMG signals in a cohort of subjects suffering from upper limb PNIs on any of the ulnar nerves, the median nerve, or the radial nerve. A protocol for surface EMG recording and intelligent data analysis was efficiently used as an examination tool for characterizing various neurological diseases, such as ALS (Chen and Zhou, 2014), stroke (Li et al., 2017), and cerebral palsy (Tang et al., 2015). In this study, we aimed to develop a non-invasive examination tool for diagnosing neurological diseases using surface EMG, which can be viewed as the first attempt to apply this to the PNI population. Its success not only facilitates PNI evaluation, but also promotes the extensive clinical use of surface EMG.

The high sensitivity and specificity in Module I demonstrated the feasibility of judging the gross arm injury as a two-classification problem, which is the prerequisite to further evaluation of injury degree for three nerves. The diversity of forearm traumatic PNI conditions is a great challenge in the design of this module. Therefore, a data-driven method was applied for mining PNI-related information using machine learning algorithms. Because of this, a general and gross judgment on whether the tested arm was injured or not can be made in this module. Unlike Module I (that uses a purely data-driven approach), Module II is largely empirical, combining anatomical and physiological knowledge. The itemized table and an accumulation deduction system did provide a quantitative evaluation of injury degrees of three individual nerves. The experimental results show that the evaluation decisions of the proposed method are highly consistent with the clinical examination results, proving the feasibility and effectiveness of the proposed method.

Some differences remain when comparing the results of the proposed method and routine clinical assessment. One arm injury (i.e., the left arm of subject 5) was wrongly judged to be normal in Module I. In addition, there were multiple cases showing slight differences in estimating nerve injury degrees by Module II as compared to clinical assessment results. For example, the left ulnar nerve of subject 3 had no injury by the clinical assessment, but our method gave it a score of eight, representing slight injury. One possible reason is the meticulous quantification process of the proposed method, which uses the more detailed ten grades than the routine clinical evaluation with only three general grades. To make a comparison between these, we had to roughly categorize the ten grades into three intervals. Therefore, there is likely to be a loss of detailed information that might account for differences in PNI evaluation. Given the rough corresponding relation, however, a higher consistency between the PNI evaluation results from both approaches was yielded when data from all subjects were pooled together. This was also due to the large tolerance of the general three-grade scale. The different mechanisms for PNI evaluation might also explain some inconsistent decisions by these methods. The clinical, electrophysiological examination focused on the integrity of the nerve conduction pathway, while the proposed method involved the evaluation of motor functions associated with the tested nerves. Considering the complexity of neuromuscular mappings in both anatomic and functional aspects, some ambiguities or differences seem reasonable. Because of this, the proposed PNI diagnosis method conformed to the physiological essence of neuromuscular function, and the quantitative evaluation decisions were found to be closer to the subjects’ motor performance, which was a better reflection of the subjects’ ability during ADL.

It is worth noting that the proposed method using surface EMG is not a substitute for the traditional clinical assessment method, but it serves as a useful and complementary tool for PNI evaluation. Furthermore, given the rapid development of mobile sensing and computing technologies, miniaturized and wearable devices make it easier to collect and analyze surface EMG signals pervasively. The intelligent expert system built with advanced signal processing machine learning algorithms makes the diagnosis convenient and suitable for family and community rehabilitation. These traits contribute to the management and treatment of PNI. The universal adaptation of myoelectric data recording and processing system can also benefit the field of forensic identification. If this system is available, there is no need to wait for a professional doctor or forensic expert to give an opinion in the forensic appraisal, and this approach can be easily operated by the grassroots police instead. Thus, this enables early judicial intervention to PNI cases and facilities the mediation of associated civil disputes. It is of great significance for Chinese law enforcement officials to quickly settle some issues with the aid of this convenient PNI evaluation tool.

Although the involuntary EMG signal following PNI mainly showed spontaneous fasciculations instead of spastic muscular activities, the proposed detection method was designed according to their same characteristics, which have been previously reported (Zhang et al., 2013a, b). Thus, the proposed method could be extended for spastic EMG detection and be applied to the detection and quantitative evaluation of upper central nervous system injuries.

It should be acknowledged that the relatively small sample size remains the main limitation of the current study. As a result, a relatively simple data analysis protocol was applied with a general purpose of verifying the feasibility of the proposed framework for quantitatively evaluating the nerve injury degree in traumatic upper limb PNI. Therefore, some detailed information cannot be considered or fully investigated in this study. For example, hand dominance usually affects the motor performance of the upper limb, and it was not considered due to the limited number of recruited subjects. The validity of the current study relies on the assumption that the effect of hand dominance is limited as compared to the impact of PNI on upper-limb motor function, which is regarded to be true in general cases. Through the cross-validation strategy, the satisfactory performance of the proposed method demonstrated its good generation and usability in actual applications (to predict the degree of PNI for an unknown subject). This finding, fortunately, confirms, in part, the limited effect of the hand dominance on the PNI evaluation. However, more data needs to be collected with an enlarged sample size to promote the usability of the proposed method, and advanced machine learning algorithms are required toward improved performance of the PNI evaluation. All of these efforts are part of our planned future work.

## Data Availability Statement

The datasets generated for this study are available on request to the corresponding author.

## Ethics Statement

The studies involving human participants were reviewed and approved by Medical Ethics Review Committee of The First Affiliated Hospital of Anhui Medical University. The patients/participants provided their written informed consent to participate in this study.

## Author Contributions

WT collected and analyzed the data, interpreted the results, and wrote the first draft of the manuscript. XZ and YS conceived the study, conducted data collection, analysis, interpretation, and substantial revision of the manuscript. BY and XiC participated in the data collection and interpretation. XuC and XG participated in data interpretation and revised the manuscript. All the authors approved the final version of the manuscript.

## Conflict of Interest

The authors declare that the research was conducted in the absence of any commercial or financial relationships that could be construed as a potential conflict of interest.
